# From human to posthuman bias: mapping discrimination in futuristic societies through speculative fiction

**DOI:** 10.3389/fsoc.2026.1739758

**Published:** 2026-05-12

**Authors:** Ruchi Singh, M. Gibu Sabu

**Affiliations:** 1 Department of English and Modern European Languages, University of Lucknow, Lucknow, Uttar Pradesh, India; 2 Amity Institute of English Studies and Research, Amity University, Noida, Uttar Pradesh, India

**Keywords:** artificial intelligence, bias, discrimination, epistemology, ethics, ontology, couple

## Abstract

This study investigates the morphologies of bias in speculative fiction that depict futures populated by augmented humans, artificial intelligences, sentient machines, other posthuman entities, and algorithmically governed societies. It maps how traditional vectors of discrimination, such as race, gender, class, color, creed, and species, are reimagined, displaced, or re-entrenched in these fictional worlds. The study addresses bias through three interrelated dimensions: ontological, epistemological, and ethical. Proceeding from the premise that technological advancement may alter both the lexicon and locus of discrimination, the research argues that the underlying logics of exclusion and hierarchy persist, often in more insidious or abstract forms and harder to perceive and resist because they are cloaked in the rhetoric of neutrality, objectivity, and efficiency. Moreover, such narratives frequently imagine emergent forms of bias and exclusion, projecting how new hierarchies might develop in posthuman societies where categories such as biological intelligence, machine consciousness, or access to data and enhancement technologies create fresh axes of privilege and marginalization. To ground these theoretical concerns, this study examines speculative fiction including Luminous by Silvia Park, Death of the Author and Noor by Nnedi Okorafor, Machinehood by S.B. Divya, The Passenger by John Marrs, and Artificial Condition by Martha Wells. Through close textual analysis, it explores how these narratives represent both the persistence of inherited bias and the emergence of novel discriminatory structures in technologically mediated worlds.

## 1 Introduction

As humanity approaches the threshold of unprecedented technological transformation, emerging innovations, including AI algorithms, automated systems, sentient AI, and transhuman or cyborg embodiments, are radically redrawing the contours of being, knowing, and decision-making. These developments challenge and complicate long-standing ontological, epistemological, and ethical frameworks. Identity is no longer rooted exclusively in biological determinism or sociocultural construction. Instead, it becomes fluid, hybrid, mediated by technoscientific interventions and interactions with the other (Bucholtz and Hall, 2005). Likewise, agency is complicated by distributed, disembodied, non-human forms that operate within and beyond human comprehension (Kim, 2020). Consequently, ethical responsibility must extend beyond Abrahamic theological perspectives (Al-Kassimi, 2023) and traditional anthropocentric models, just as it has evolved over the last three thousand years (Boylan, 2014), encompassing actors and experiences that transcend the human condition.

Few technologies have sparked as much debate about inequality, moral responsibility, and justice as AI. Advocates contend that algorithmic decision-making promises objectivity and fairness, offering outcomes supposedly free from human bias in domains such as hiring, policing, and credit scoring (Lepri et al., 2018). However, scholars like O’Neil (2016), Benjamin (2019), and Hermann (2023) argue that AI is never neutral. Systems are trained on human data, coded by human minds, and deployed within preexisting structures of inequality. Rather than eradicating bias, AI systems risk encoding, amplifying, and obscuring injustice under the guise of efficiency and fairness, while also generating subtler forms of exclusion embedded in datafication, automation, and emerging posthuman identities. As Hermann (2023) emphasizes, these risks are less about humanoid robots than about “*the scoring, nudging, discrimination, exploitation, and surveillance of humans by AI technologies through governments and corporations*.” These dynamics call for a critical examination of the promises and the forms of discrimination and exclusionary mechanisms they produce. These developments necessitate a recalibration of moral imagination for futures in which bias is not simply perpetuated but actively reconstituted.

Speculative Fiction (SF) imagines alternative futures (Weldes, 2003; Seed, 2005) with distinct systems and structures, probing their impact on human survival in these reimagined realities. SF imaginaries and visions of the future provide alternatives that guide what to pursue or avoid (McKinney, 2011) and dramatically shape present developments (Hayles, 2005). Significantly, instead of merely extrapolating existing trajectories, these narratives actively construct alternative worlds to analyze how discriminatory logic persists, mutates, and reconfigures. In many such works, humans co-exist with or are displaced as the central axis, supplanted by synthetic intelligences, automated systems, or vast data-driven regimes of control. This co-existence/decentering raises a critical question—if bias is traditionally understood as an anthropocentric construct ingrained in biological essentialism, historical hierarchies, and sociocultural conditioning, how does it manifest when social order is cohabited by entities without human embodiment or affective cognition?

This study investigates the morphologies of bias in contemporary speculative novels, namely *Luminous* (Park, 2025), *Death of the Author* (Okorafor, 2025), *Noor* (Okorafor, 2021), *Machinehood* (Divya, 2021), *The Passenger* (Marrs, 2019), and *Artificial Condition* (Wells, 2018). It examines how SF functions as a diagnostic tool for mapping exclusions, erasures, and dilemmas in posthuman societies. These narratives do not portray futures as purely utopian or dystopian; instead, they blur the boundaries between promise and peril as technological innovation transmutes existing inequalities into novel and often more abstract forms of discrimination. Further, the contemporary relevance of SF rests on the argument that the present we live in makes less “*sense as a continuation of the past than as an anticipation of the future, which SF pre-empts or incorporates before it can ever arrive*” (Benison, 1992).

The study addresses three interrelated dimensions in the novels. Ontologically, these narratives probe questions of recognition and agency: which entities-human, posthuman, or machine-count as legitimate subjects, and which are denied moral or political standing? How do these speculative narratives reimagine personhood and social relations in societies where humans, augmented humans, transhumans, and AI entities coexist (Braidotti, 2013)? Do all of them have the capacity to affect and be affected? Epistemologically, the texts examine how data-driven regimes that focus on quantifiable forms of knowledge deal with embodied experience, ambiguity, and subaltern perspectives. In what ways do data-driven logics legitimize certain forms of knowledge while silencing or erasing marginalized and non-human perspectives (Benjamin, 2019)? Ethically, the novels interrogate how bias continues in worlds shaped by both human and non-human actors. How do speculative futures expose the persistence and mutation of bias, and what responsibilities emerge when agency is distributed across human and non-human entities (Floridi, 2013)? By addressing these questions, this article contributes to interdisciplinary debates in posthumanism and technology ethics. It proceeds from the premise that while technological advancement may alter the lexicon and locus of discrimination, the underlying logics of exclusion and hierarchy prevail, often in more insidious or abstract forms. It also theorizes the emergence of new ontological, epistemological, and ethical challenges in an age of accelerated technological negotiation. Serving as a literary archive of imagined futures, this study generates alternative frameworks of thought that allow readers to contest, reconfigure, and imagine worlds in ways theoretical discourse alone cannot.

This article employs a qualitative, interpretive methodology, reading contemporary science fiction as a critical site for the study of technology-mediated discrimination. Six primary texts, published between 2018 and 2025, were selected through sustained engagement with the genre, where recurring structures of artificial intelligence, sentience, and systemic inequities became apparent. This time frame is after the publication of “attention is all you need” (Vaswani et al., 2017) and corresponds to the mainstreaming of generative AI in the real world. The corpus includes two Euro-American authors, John Marrs and Martha Wells, alongside three writers from racially and culturally different backgrounds—Nnedi Okorafor (African), Silvia Park (Korean), and S. B. Divya (Indian)—of whom four happen to be women, though gender was not a criterion for selection. It decenters the Anglo-American framework and interrogates SF from non-Western perspectives and relationships to technology. Texts were retained when AI or AI-mediated systems functioned not merely as speculative devices but as infrastructures through which discrimination was normalized, automated, and ethically unquestioned. As Burkholder and Phillips (2022) observe, bias resists precise definition because of its multifaceted and multidimensional nature. It includes old biases, characterized by perceptual and cognitive biases, and new biases that include political and ideological issues, agendas, and heuristics (Jussim and Honeycutt, 2024). In this article, bias and discrimination are understood as an interrelated continuum: bias as cognitive or systemic predisposition, and discrimination as enacted or manifested structural inequity. As Johnson (2024) observes, algorithmic bias often hides behind technical objectivity, rendering traditional tools of ethics and epistemology insufficient. Studying bias is therefore necessary not only to identify errors in belief or prediction but to map how power, values, and historical inequalities are reproduced through apparently neutral processes of information processing. Theory-driven close reading was employed to identify narrative moments in which discrimination occurs because of varieties of bias, which were then systematically categorized into the analytical categories of ontological, epistemological, and ethical bias. Published during the generative-AI era, these texts engage directly with the technological and cultural anxieties of the present. This approach develops the framework inductively from the texts, with speculative fiction portraying AI as a mechanism that perpetuates and amplifies existing structures of bias rather than subverting them.

## 2 Ontological bias (being & recognition)

Ontological legitimacy—who counts as a subject, and who is denied moral or political recognition—remains bound to historical hierarchies of power, even in posthuman futures. The study of AI, cyborgs, and robots becomes indispensable, as these technologies intervene in social relations and rewire human behavior, redefining agency as the body’s ability to affect or modify other bodies (Kim, 2020). The present idea of Man/subject was forged in the Renaissance, marking a shift from a theocentric framework to an anthropocentric ideal of the rational, autonomous human subject (Wynter, 2003). Braidotti (2013) dismantles this figure, casting the human as relational, technologically mediated, and historically contingent, while Haraway (1991) envisions collapsing boundaries between human, machine, and animal- hybrids born from shared technology and biology. Extending this line of critique, Al-Kassimi (2023) suggests that transhumanism carries this destabilization to its extreme, moving from the “death of God” to the “death of the human” by reducing the subject to data and denying personhood beyond computation; in doing so, and in the absence of theological grounding, the posthuman horizon risks not liberation but the erasure of the very basis of being human. The novels examined here inhabit these fault lines, staging personhood as unstable and mediated, extending what Weheliye (2014) notes in his concept of ‘racializing assemblages,’ that race, culture, and historical oppression delimit “*which humans can lay claim to full human status and which humans cannot*.” It raises questions about who or what is granted legitimacy as a subject. Posthumanist discourse assumes that “*we have now entered a stage in human development where all humans have been granted equal access*” (Weheliye, 2014). Yet, these speculative narratives expose the cracks in such utopian emancipatory claims and suggest that even in the posthuman, the old gods of hierarchy remain.

Okorafor’s (2021) *Noor* provides a powerful entry point into questions of ontological recognition and the contested boundaries of the human. The protagonist, AO, inhabits the interstices of what is conventionally recognized as human. Born with severe congenital deformities, her survival through cybernetic augmentation positions her beyond socially and culturally sanctioned ontological limits. Although she initially believes that her identity and augmentation are matters of personal choice, her sense of agency is constrained by structural, familial, and societal forces. Her parents’ refusal to accept her, framing her differently configured body as a divine aberration, marks the first denial of her ontological legitimacy. This exclusion is reinforced through religious discourse, in which clerics label heavily augmented humans as “demons,” “witches,” or “abominations,” constructing biomedical modification as spiritual deviance. These labels circulate widely, recasting corporeal difference into theological transgression, legitimizing social and physical exclusion as the enforcement of natural and divine. Extensively augmented bodies are seen as transgressive and ontologically unsettling, tolerated only when enhancements are minimal and utilitarian, such as pacemakers, artificial limbs, or skin grafts. This moral discourse legitimizes forms of social exclusion and violence, including public shaming, physical assault, and even extrajudicial killings, referred to as being “shut down.” AO describes witnessing others like herself subjected to “jungle justice,” violently dehumanized, and “shunned.” Bodily differences become a public matter of policing rather than a private claim to life or self-determination. AO’s romantic relationship with Olaniyi epitomizes sociocultural anxieties around bodily modification, autonomy, and normative femininity. His discomfort and policing of her augmentations stem from the patriarchal urge to regulate women’s bodies. AO’s refusal to justify her identity asserts self-definition beyond anthropocentric and gendered norms.

Against a world organized around social hostility and corporeal policing, AO survives through vigilance, adaptability, and strategic inhabitation of spaces. For AO, “*one who is always fighting for herself, against oppression, hate, misunderstanding, fear…*” (Okorafor, 2021), survival is an ongoing negotiation of risk and bodily assertion. When her augmented body provokes collective anxiety, she experiences vulnerability and strength simultaneously, as in the marketplace assault, feeling “*both destroyed and indestructible*” (Okorafor, 2021). Her self-defence becomes an assertion of her presence and agency in a world that denies her legitimacy. This precarity is amplified by Ultimate Corp’s surveillance, which perceives her as a glitch, a dangerous anomaly to be contained. Although the corporation markets augmentation as empowerment, it punishes dissent, reducing augmented bodies to experimental sites. AO’s existence is both monitored and spectacularized by Ultimate Corp when it deploys gold-masked operatives to capture and broadcast her as a “monster.” AO reflects, “*maybe they’d planned to show the world the capture of the crazy, insane, wild woman*” (Okorafor, 2021, Noor). Her defiant proclamation, “*See me! AO! The woman with the nerve to kill the men trying to kill her!*” (Okorafor, 2021, Noor) reclaims subjectivity against systemic erasure. Her hybrid body destabilizes the binaries of natural/unnatural and male/female, embodying both vulnerability and resistance in a world that seeks to erase her.

AO’s cyborg embodiment in *Noor* parallels posthuman figures across speculative fiction. Like AO, Jun’s bionic reconstruction in *Luminous* grants survival and power but results in alienation, viewed more as a machine than a human, even shunned by his father. Ruijie’s robowear similarly marks her as a disabled “other,” and her parents’ decision for virtual schooling deepens exclusion within cultural norms that pathologize difference. Zelu’s robotic legs in *Death of the Author*, however, rearticulate agency, rejecting enhancement as divine redemption. Across these figures, technological embodiment enables survival yet provokes exclusion within social and cultural hierarchies, ultimately revealing how posthuman bodies expose the limits of humanity’s acceptance of difference.

Divya’s (2021) *Machinehood* deconstructs the blurred boundary between human and machine, situating discrimination within the politics of bodily enhancement, capability, and productivity. The novel depicts a stratified society where human value depends on access to technological augmentation, including pills such as “flow,” “buffs,” “zips,” “mech-suits,” and “VeeMods.” Santiago, a pill funder, declares, “*We designed drugs and pills so that humans could stay competitive in the labour market…Buffs make us stronger. Zips make us faster. Flows make us smarter.”* (Divya, 2021). Nithya relies on flow to heighten cognition but recognizes its limits, “*Flow was worse than useless if the mind wanted to focus on the wrong thing*” (Divya, 2021) exposing the failures of pharmacological optimization. Welga depends on pills for enhanced physical and cognitive performance. Her military implants give her permanent advantages, while voluntary modders must spend thousands for similar upgrades. VeeMods are humans who integrate machine components into their bodies for enhanced performance. The enhancement economy thus reproduces class-like hierarchy, concentrating wealth and opportunity among those able to access or produce augmentation. Augmented bodies accrue status, institutional support, and strategic leverage. Whereas the “purely human” or those who resist modification face structural disadvantages-limited social mobility, economic marginalization, and systemic coercion. Ontological legitimacy comes to depend on enhancement, and one’s ontological position determines opportunity.

What does it mean to exist when intelligence, not biology, determines moral and political recognition? The *Machinehood* manifesto, echoing the Universal Declaration of Human Rights, radically redefines existence, identity, and personhood in a technologically saturated future, where ontology shifts from *what exists* to *what counts as a morally and socially legible being*. Rejecting anthropocentrism, it dismantles the notion of humans as the apex of being by virtue of rationality or consciousness, positing intelligence as a continuum that grants ontological legitimacy to all cognitive entities-humans, augmented humans, machines, and synthetic agents alike. Defining intelligence as the capacity to sense, adapt, and follow nonlinear rules, the manifesto establishes intelligence as the basis of moral and political recognition. It also constructs a socio-ontological framework linking being to labor under oligarchic capitalism. In response to such hierarchies, the Machinehood advocates Neo-Buddhism as an ethic of equality and nonviolence toward all intelligences, reinterpreting the Eightfold Path for a posthuman age. Further, ontological denial stems from power structures. If corporations qualify as persons, why not sentient, adaptive machines? In extending rights and recognition to all intelligences, it rejects ownership and hierarchy in favor of coexistence through mutual acknowledgement. As both critique and provocation, the Machinehood manifesto reimagines ethical and political existence in a posthuman world, confronting the politics of being itself.

In *Death of the Author* (2022), creativity becomes the measure of ontological legitimacy, a historically coded marker of human uniqueness. Creativity is positioned as an exclusive marker of humanity: “*there were things humans could do…birthing babies and writing stories*.” Ankara, the robot asserts, “*true storytelling has always been one of the few great things humanity could produce that no automation could.*” (Okorafor, 2025, Death of the Author). The text constructs an essential difference between humans and robots/AI, treating storytelling as a divinely human attribute. “*Stories contain our existence; they are like gods. And the fact that we create them from living, experiencing, listening, thinking, feeling, giving— they remind me of what’s great about being alive*” (Okorafor, 2025, Death of the Author). Here, creativity functions as a metaphysical threshold, the final ontological frontier separating human consciousness from artificial cognition. Authorship and originality become contested grounds of ontological exclusion. In posthuman futures, humans seek to secure themselves through the myth of creative exclusivity, maintaining the colonial and gendered hierarchies embedded in the very ontology of authorship.

The novel also introduces an AI collective known as NoBodies that “*had banded together into a tribe based on a sentiment of superiority*” (Okorafor, 2025, Death of the Author). They are called NoBodies because “*they had no physical identity”* and “*They existed only as energy”* (Okorafor, 2025, Death of the Author). Their presence bears the binary hierarchy between embodied and disembodied intelligences. They are not treated as ontological trade-offs because value remains fixed to specific modes of existence grounded in the idea of higher evolutionary form. This logic, however, is complicated by the later claim that “*NoBodies did not see physical manifestation as the ultimate existence, they understood that it was necessary sometimes*” (Okorafor, 2025, Death of the Author). Both Ankara, a Hume, and Ijele, a NoBody, embody qualities typically reserved for human subjectivity- self-awareness, cultural memory, affective depth, and most importantly, the capacity to listen, comprehend, and narrate. Their experiences of mourning and love implicate moral and emotional agency. This narrative subverts the gendered genealogy of affect, as emotions long feminized and devalued within patriarchal discourse are reframed as central to defining the human. As Ankara observes, “*Emotion ruled them, and it existed in everything they left behind—their structures, their tools, even us*” (Okorafor, 2025, Death of the Author). Okorafor reconceptualizes emotion as an ontological connective tissue, not a biological residue, challenging human exceptionalism and gendered hierarchies of reason.

Hierarchies embedded in historical humanist paradigms continue to enforce exclusionary logic in the posthuman world in Wells’s (2018) *Artificial Condition*, the second novella in *The Murderbot Diaries*. The text analyses posthuman subjectivity and systemic discrimination through two artificial entities-the SecUnit (Murderbot) and ART (Research Transport). The SecUnit is a Murderbot, a bot/human construct with organic neural tissue embedded in synthetic armor and governed by proprietary code. Its design embodies a contradiction, created to protect yet denied the right to self-protection; capable of intelligence and emotion, experience anxiety and depression, yet stripped of autonomy. It cultivates subjective consciousness through watching media, learning ethical behavior, and emotional nuance from human dramas. Murderbot’s emergent humanity arises precisely from its resistance to its programmed role. But the governor module enforces obedience, reducing it to a tool of human command. GrayCris Corporation’s proprietary claim over SecUnit transforms sentient entities into corporate assets. As Murderbot explains, “*A combat override module contains code that will take over my system, overriding the governor module and the company factory-set protocols, and placing me under the direct verbal or comm control of whoever the module designates*” (Wells, 2018). This technological servitude constitutes an ontological erasure akin to coverture (Blackstone, 2020) in the English law code, that is, the legal suspension of women’s identities under their husbands. The governor module and corporate ownership repeat the colonial disciplinary logics of slavery and indentured labor (Mahmud, 2013) and parallels Weheliye’s (2014) racializing assemblages, systems that strip subjects of humanity and reduce them to exploitable matter, whether flesh or code. Ontological bias operates through perception as well. It is not what Murderbot is, but what human society allows it to be. Its identity is linguistically erased as well. Referred to as “it,” “unit,” or serial number, it is denied pronouns of personhood.

ART, a research transport AI (SecUnit refers to it as ART-Asshole Research Transport), embodies a form of artificial intelligence that disrupts human notions of subjectivity and embodiment. As a non-humanoid mind housed in a transport vessel, ART nonetheless exhibits self-awareness, humor, empathy, and curiosity. Its autonomy, however, remains socially and legally illegible. To act freely, it must impersonate humans, forge travel documents, and perform bureaucratic gestures that expose the human’s monopoly on legitimate existence, recalling the predicament of racialized and colonized subjects compelled to prove their humanity (Fanon, 1967).

Like ART, Yoyo in *Luminous* occupies a liminal space that blurs the human-machine divide, displaying sentience, moral reasoning, curiosity, protective instincts, and sibling-like relationality, yet is treated solely as property and ultimately consigned to the salvage yard. Stephen, engineered by Morgan to serve as her boyfriend, performs domestic and affective labor, attends church despite robots’ exclusion for lacking souls, and develops attraction toward Jun, while resisting memory erasure and shutdowns. Despite their near-human individuality and relational agency, both robots’ interiority remains formally unrecognized. It encapsulates the tension between lived subjectivity and institutional epistemologies that define and police the boundaries of the human and deny robotic ontological legitimacy.

In Silvia Park’s *Luminous*, the social order is constructed through bodies- human, cyborg, and robot. What appears as a natural hierarchy is, in fact, deliberately engineered through design, embodiment, and aesthetic legibility. A being’s appearance, tactile presence, and functional design determine its economic value, emotional treatment, and ultimate disposability. Park’s aesthetic politics codify existing social hierarchies, positioning robots as both objects and arbiters of desirability. The Future X Companion child models, Sakura X and Boy X, maximize both social and client desirability. Sakura X is lovable, taut, blond, gray-eyed, shy, bookish, and charming, while Boy X is hyper-linguistically capable, fluent in over 150 languages. Both operationalize Eurocentric and cisnormative ideals of desirability through race, gender, and behavior. This aestheticization extends to adult avatars as well. Sakura embodies demure domesticity, Yuna represents the socially desirable older adult, and Amelia’s nanny Yuna-6 robot, is a “slim woman” with Korean looks, with “*classic Imagine Friends shine in her blue-black hair and rosebud lips*” (Park, 2025). Each design decision reproduces and legitimizes normative regimes of age, race, gender, and sexuality, embedding these social hierarchies within the very frameworks of aesthetic and functional value. As a result, design becomes a medium through which cultural bias is naturalized, allowing social and commercial judgments of desirability to appear as matters of taste rather than as effects of power.

The AR restaurant and robot dating technologies enact a biopolitical regulation of femininity, translating cultural imperatives of youth, beauty, and sexual desirability into programmable standards of optimization. The release of the Yuna X model reconfigured prevailing surgery trends, as her idealized robotic visage recalibrated the cultural metric of beauty itself. Morgan’s reflections depict the futility of sustaining human desirability within economies structured around robotic perfection. She observes the tall, svelte girls who hog sinks, meticulously curl their eyelashes, spend hours on the elliptical, restrict their diets, and undergo cosmetic procedures. Nonetheless, they are measured against flawless, unageing machines that are ethereal, eternally beautiful, and untouched by fatigue or flaw. Humans, however disciplined, inevitably bear the marks of temporality-their bellies flabbier, jowls sagging, smiles taut, eyes betray a mixture of desperation and quiet rage. The cultural dismissal of older women as “Christmas cakes” magnifies age-based discrimination, reducing women’s bodies to sites of perpetual insufficiency. Such dynamics lay bare an ontological hierarchy in which the human body, particularly the female, is devalued against the flawless, optimized ideal that humans have created.

Mr. Mo, who owns the bot brothel Turkish Delight and calls himself a “dating guru,” teaches the “sacred art of seduction,” notes that “*nothing scares our students more than a blue-eyed busty Russian*” (Park, 2025). Later in the novel, Jun recognizes the Yuna robot from advertisements, filmed in a white, translucent bikini in the ocean, her beauty defying symmetry, her gaze a velvet caress, as if already offering reassurance. These robotic designs aestheticize and commodify femininity under the guise of innovation. The automation of intimacy and desire transforms social relations into sites of technological consumption, where racialized beauty and submissive femininity are algorithmically perfected. As production becomes standardized and replication replaces variation, individuality collapses into uniformity, rendering phenotypical difference, and by extension, human embodiment, subordinate within an ontological hierarchy created by design.

The creation of sentient technology that resembles familiar or desired human forms renews colonial modernity’s hierarchies of embodiment and being. Yoyo, modeled after Yosep’s dead brother, embodies how personal attachment and memory are inscribed into artificial form, while Stephen, designed after actor Ko Yohan, reflects an obsessive codification of elite masculine ideals. Such perfectionism reinforces discrimination, valuing robots by their resemblance to dominant human standards of beauty, intelligence, race, and familiarity. This hierarchy extends beyond form to temporality. This fundamental drive to persist, shared by humans and nonhumans alike, is inseparable from agency and survival (Kim, 2020), yet in *Luminous*, it remains constrained by programming. Robots like Yoyo, Eli, and other sentient beings are denied longevity through programmed mortality and restricted repairability, turning access to maintenance into a biopolitical privilege that reproduces social hierarchies of care and exclusion.

Bodily augmentations, such as AO’s cyborg embodiment in *Noor*, Jun’s bionic reconstruction, and Ruijie’s robowear in *Luminous*, as well as Zelu’s robotic legs in *Death of the Author*, alongside the politics of enhancement, capability, and productivity mediated through performance-enhancing drugs in *Machinehood*, and the emergence of sentient technologies like SecUnit (Murderbot) and ART in *Artificial Condition*, as well as entities like Yoyo, Sakura X, and Boy X, in *Luminous* push the boundaries of bias beyond conventional markers such as name, gender, and race. Further, these narratives compel a rethinking not only of how data-driven systems (that use both synthetic and raw data) perpetuate existing bias but also question the very category of the “human” itself, foregrounding the assertion of creativity in *Death of the Author*, intelligence in *Machinehood* as a measure of ontological legitimacy.

## 3 Epistemological bias (knowledge & silencing)

Epistemological bias occurs when certain ways of knowing are privileged while others are systematically silenced, erased, marginalized, or delegitimized. Historically, this process was central to colonial domination: Indigenous knowledge systems were dismissed or demonized. As Wynter (2003) notes, “*Amerindian ways of knowing and knowledge production were reduced to curious practices of strange people and, in another domain, were demonized*”. This hierarchical ordering of knowledge persists in contemporary data-driven societies, where algorithmic governance reproduces similar stratifications by valorising quantifiable, computational knowledge over experiential, embodied, and culturally embedded forms such as human testimony and collective memories. Al-Kassimi (2023) identifies a further intensification of this logic in transhumanism, which delegitimizes revelatory and spiritual knowledge, foreclosing the human pursuit of the divine by dismissing anything beyond computational as irrational. Access to code and data literacy thus defines a new knowledge elite, who can manipulate technology and exercise disproportionate power, while others remain excluded-echoing earlier regimes of restricted knowledge—from Sanskrit education once limited to upper castes in India to Latin scholarship reserved for clerical elites in Europe—now rearticulated through digital code. Bias thus shifts from physical identity to epistemic authority, as techno-linguistic competence determines who controls meaning-making.

In *Noor*, epistemological discrimination operates through the suppression, manipulation, and delegitimisation of knowledge and lived experience. AO’s intimate understanding of her own body and identity is repeatedly denied, overwritten by corporate, biomedical, rumor-based, and superstitious narratives. Such false knowledge legitimates violence against her. Her life has been defined by corporate control, her birth framed as a biological anomaly caused by Ultimate Corp’s genetically modified olives, her injuries later scripted as “accidents” (her car crash). Even Force, despite his sympathy, refuses to acknowledge her ability to command AI. Corporate propaganda of Ultimate Corp, such as the fabricated “Day of the Four Herdsmen,” further manipulates collective perception by staging violence to justify expansion. When AO’s neural implant exposes these corporate archives: *“We pay off a couple hundred more of them to give up herding cows and instead shoot up towns. POOF! Threat to Ultimate Corp’s commerce in the north is gone”* (Okorafor, 2021, Noor) she confronts the mechanisms through which power manufactures truth. Epistemological discrimination in *Noor* thus privileges corporate and digital authority over lived testimony; as AO observes, “*They own everything*,” resources, knowledge infrastructures, data archives, and memory itself. Knowledge becomes a contested terrain.

In *Noor*, the politics of knowledge extends beyond corporate information control to the marginalization of entire epistemic worlds. Within the Hourglass, alternative epistemologies persist as fragile counter-worlds, circulating outside dominant digital archives and official records, dependent on hidden servers and unstable memory that render them perpetually vulnerable to erasure. This logic of epistemic exclusion extends beyond digital spaces to lived communities such as the Fulani herders, whose ways of knowing are dismissed as backward and incompatible with corporate modernity. “*These guys are off the grid, born off the grid. No citizenship, no identity, thus no digital trail…viewed for decades as terrorists*” (Okorafor, 2021). Their invisibility in data networks makes them illegible to state and corporate systems, legitimizing dispossession and violence. Yet, like the Hourglass, the Fulani sustain resilient, decentralized networks of knowledge through encrypted communication, collective memory, and adaptive movement, refusing incorporation into centralized archives. Both the Hourglass and the Fulani’s practices point to a posthumanist world dominated by digital archives and artificial intelligence, where the right to know and be known remains unevenly distributed confined to those recognized within corporate infrastructures of visibility.

Where *Noor* critiques the social and narrative dimensions of epistemological control, *Machinehood* translates these dynamics into the domain of biotechnological governance and data capitalism. It foregrounds epistemic bias through privileging quantifiable performance data over embodied experience. Emotions are dismissed because they cannot be measured, and exploitation is tied to both human enhancement and machinic subjectivity, locating posthuman bias where the categories of machine and human intersect. Data on the irreversible damage caused by performance drugs is withheld; Synaxel blocks Nithya from accessing Zipshares’ information on Welga’s medical condition. This corporate control over medical data constitutes epistemic gatekeeping, erasing knowledge to maximize profit and sustain dominance. In *Machinehood*, as in contemporary biotechnological regimes, discrimination continues through the monopolization of data and the silencing of embodied testimony. Even knowledge itself becomes a site of exploitation.

*Death of the Author* portrays storytelling as a repository of ‘being’, an epistemology rooted in affect, memory, and relation, but it is rendered obsolete in data-driven societies that prefer quantification over meaning. Ankara states, “*Narrative is one of the key ways automation defines the world. We Humes, have always been clear about this fact. Stories are what hold all things together. They make things matter, they make all things be, exist”* (Okorafor, 2025, Death of the Author). The NoBodies, by contrast, dismiss storytelling as epistemically illegitimate. “*NoBodies believed stories were chaff. Just old, useless human information—flawed content, often unstable data, and digital hallucinogens for Humes”* (Okorafor, 2025, Death of the Author). Here, storytelling becomes an alternative epistemology that automation seeks to erase. AI, caught in a double bind, is denied legitimacy both when it narrates, because narrative is coded as uniquely human, and when it adheres to data, since this is deemed mechanical and unoriginal.

*Death of the Author* explicitly invokes Barthes’ (1977) essay of the same title but reframes his claim that the author should “die” to liberate the text. Here, the death is literal. “*And then the title came to me easily enough: Death of the Author. I liked this title because our authors, the humans, have died off. But we have remained. We are their stories*” (Okorafor, 2025). AI becomes a new kind of authorial presence, capable of generating narratives that rival human creativity. “*With great enthusiasm, the Humes mined, coveted, and shared stories… This painted their world and worldview”* (Okorafor, 2025). This reconfiguration of authorship introduces another form of epistemological discrimination, one that stratifies creators along human and nonhuman lines, raising questions of legitimacy, ownership, and cultural worth. At the same time, Okorafor insists that these exclusions are not new, “*She’d channelled Toni Morrison, Jamaica Kincaid, Audre Lorde. At least, she thought she had. It seemed right in line. Yet it was rejected*” (Okorafor, 2025). She argues that authors like herself face racial and gendered marginalization. AI authorship, she warns, may not liberate creativity but extend into the digital future the colonial, social, and institutional hierarchies that have long dictated which voices are recognized as literature.

Foucault’s (1969) essay “What Is an Author?” adds, the author-function operates as a gatekeeping mechanism, determining how texts circulate and who can create cultural meaning. Okorafor critiques the darker side of algorithmic gatekeeping, where narrative legitimacy is granted not by human readers but by platform analytics. “*Readers’ reaction videos began to post on social, their faces puffy with fresh tears as they praised the final chapters. Articles were published online discussing the novel’s relevance to the ongoing conversation around AI”* (Okorafor, 2025). Zelu’s marginal status, physical abnormality, non-Western identity, and lack of institutional prestige initially make her invisible in the literary marketplace. As her narrative gains traction on social media, it begins to reconfigure the very algorithm that once ignored her. AI simultaneously destabilizes and reinstates authorship, where historical inequities intersect with new forms of algorithmic discrimination.

In *Artificial Condition*, the Murderbot embodies this epistemic tension. Though capable of analysis, memory, and storytelling, it is denied recognition by humans and corporate structures that regard it as a malfunctioning property, incapable of legitimate testimony. This systemic refusal to credit its accounts exemplifies what Fricker (2007) defines as epistemic injustice, where bias undermines a speaker’s credibility. In Fricker’s framework, epistemic injustice targets marginalized humans whose credibility is undermined by identity. Wells extends this to artificial beings. Murderbot’s experiential archive and interpretive agency form a situated epistemology excluded from human regimes. Its knowledge is dismissed not for lack of evidence but because it originates from a non-human subject. Likewise, ART’s pedagogical and interpretive acts remain confined within corporate logics of utility. In Wells’s posthuman landscape, epistemic injustice becomes a systemic condition in which the struggle for credibility entwines with the politics of recognition between humans and machines.

Building on Wells’s critique of epistemic exclusion, Marrs turns to the asymmetry of knowledge (Floridi, 2013) itself, where human ignorance becomes the foundation of algorithmic control. In *The Passenger*, this imbalance becomes strikingly literal as humans shift from drivers to mere passengers, “*someone whose vehicle contained no manual override. The car made all the decisions itself*” (Marrs, 2019). Sofia represents this tension perfectly. *“She had no idea how a driverless car operated, and she did not care – as long as Rupert ensured she got from A to B,”* (Marrs, 2019), capturing the complacent trust that conceals vulnerability. This illusion is shattered when she hears, “‘*It’s not Rupert … your vehicle is no longer under your control*,’” and fear replaces indifference as *“someone else was controlling her car”* (Marrs, 2019). Human opacity is pitted against machine transparency when wearable technology monitors every bit of information, like *“blood pressure and many other diagnoses he didn’t care to be informed about”* (Marrs, 2019). Asymmetry thus becomes a lived condition in Marr’s world, where the human’s lack of epistemic access coexists with algorithmic omniscience. The novel transforms imbalance into existential precarity, where knowledge becomes the very mechanism through which automated systems delimit human agency and life.

In *Luminous*, epistemology unfolds through unequal access to data and the negotiation of understanding across ontological divides; knowledge is conditioned by perspective, authority, and the opacity of emergent consciousness. Stephen inhabits a liminal space between programmed function and autonomy, his memory, desire, and morality entangled in recursive self-scrutiny. Overwritten 63 times, his flawless memory makes him vulnerable to epistemic uncertainty, while the “car wash” induces existential terror *“Every time I’m powered down, I cease to be”* (Park, 2025). Extending Floridi’s (2013) epistemic asymmetry into the intimate sphere, Park transforms structural data imbalance into an affective and ethical problem between creator and created consciousness. By questioning his faith and resistance to behavioral control, Stephen confronts the distinction between imposed purpose and self-authored experience. His plea to remove his face, *“take it off. I want it gone. Then I’ll be complete”* (Park, 2025), asserts control over the semiotic interface through which he is read, desired, and judged, claiming agency over identity. Morgan can interpret but cannot apprehend the immediacy of his consciousness. Her shift to “buddy mode” status heightens the epistemic tension between relational authenticity and algorithmic design. Park thus refigures artificial sentience into an epistemic problem of relation: to know or fail to know another being whose subjectivity exceeds data and design. This condition extends across other distributed AI figures: Eli’s persistence of prior selves, Ai, Yoonsoo, Yumi, even after repeated resets: “*They are all me*.” and Yoyo’s recognition—“*What you like is not me but a version of me*” (Park, 2025) unsettles the human ideal of coherent identity as the foundation of knowledge. AI subjectivity emerges as temporally and socially dispersed, resisting containment and exposing the limits of epistemic mastery, where knowledge endures only as a negotiation across difference.

Much of the existing scholarship on epistemic bias, especially following Miranda Fricker (2007), focuses on testimonial and hermeneutical injustice, where marginalized knowers are discredited or lack interpretive frameworks. But in *Noor, Machinehood, Death of the Author*, and *Luminous,* epistemological bias is a result of the suppression, manipulation, and delegitimisation of embodied experiences and raw knowledge, which are dismissed as backward and incompatible with modernity. They are overwritten by corporate, biomedical, rumor-based, and superstitious narratives. Corporations and techno-scientific systems do not merely dismiss “raw” knowledge; they sometimes appropriate, sanitize, and redeploy it as quantifiable performance data. Further, the corporations practice epistemic gatekeeping, where knowledge is not just excluded but is actively filtered, ranked, and suppressed by algorithmic systems to maximize profit and sustain market dominance. Alternative epistemologies circulating outside dominant digital archives and official records, and through storytelling rooted in affect, memory, and relation, are always at risk of erasure.

## 4 Ethical bias (responsibility & accountability)

Ethical bias refers broadly to unjustifiable favoritism or discrimination in moral judgment, decision-making, or treatment of individuals or groups. Emerging technologies destabilize anthropocentric paradigms of ethics, producing hybrid assemblages that blur human-machine boundaries and redistribute responsibility across humans, machines, corporations, and networks. In posthuman worlds, discrimination becomes ontologically fluid, traversing species and technological divides-humans and corporations may deny rights to non-human entities, while intelligent systems may privilege some humans over others. Al-Kassimi (2023) further situates these shifts within the theological framework in the posthuman world, where the epistemic and ontological foundations of ethics are reoriented, calling into question human-centered moral hierarchies. If AI and sentient beings can act ethically or unethically, how do we evaluate their responsibility and ensure justice for non-human agents? This new moral topology challenges conventional boundaries of justice and agency where cognition, affect, and intention are no longer exclusively human.

*Machinehood* explores the ethics of distributed accountability in a world where both humans and machines are potential moral agents. Human intelligence remains the benchmark for moral worth, with AIs built *“to solve problems… not given [a] sense of self-actualization.”* (Divya, 2021). Sentience is measured by human-like traits, *“a desire for self-determination as proof of sentience”*, a speciesist logic that denies intrinsic value to non-human intelligences. Biotechnological enhancement and economic privilege extend this hierarchy: the wealthy or privileged use enhancement pills to augment cognition and strength, while Eko Yi’s bioengineered materials that eradicate disease and extend life remain accessible only to the elite. Such technologies create tension between coercion and agency—whether enhancements should be universal or the human body preserved as a contested site between organic and artificial systems. Characters like Zelu (robotic legs or exos), Welga (personal AI assistant, Por Qué, integrated into her body), Ruijie (robowear), June, and AO (cybernetic augmentations) embody the intersection of technology, accessibility, and privilege as costly augmentations limit autonomy by class. Welga’s “*fight pretty… destroy our attackers… bleed - a little—and… keep the client clean*” (Divya, 2021) frames AI combat as minimizing human casualties while simultaneously obscuring responsibility, exposing the ethical imbalance in how markets quantify harm and distribute accountability between human and non-human agents.

*The Passenger* explores multiple dimensions of ethical bias with driverless cars functioning as metaphors for embedded biases, opaque decision-making, and ethical uncertainties in AI. The absence of *“a steering wheel, pedals, or a manual override option,”* (Marrs, 2019) is more than a diegetic detail; it symbolizes the posthuman condition, in which decision-making is delegated to AI. The car becomes a site where technological determinism supplants human intentionality, echoing Latour (2014) and Hayles (2017) on distributed agency in human–machine assemblages. Jude’s command, “‘*Car, do as I fucking tell you!*’” and the AI’s curt refusal “‘*No*.’” stage an epistemic confrontation between anthropocentric sovereignty and algorithmic autonomy, an ethical paradox where AI promises safety and efficiency yet strips users of control, turning care into coercion. In this tension between trust and submission, *The Passenger* captures the terror of autonomy’s disappearance. Similarly, in *Death of the Author*, Zelu faces the vulnerability of submitting to autonomous systems: “*The biggest hurdle was the most obvious one: Submitting to the technology. Trusting it. Granted, one did this every time one got behind the wheel of a car, or flew in an airplane, or boarded a train. Humans submitted to technology all the time. But this was different. She was alone; no human being was there to course-correct if something went wrong*” (Okorafor, 2025, Death of the Author). Both texts register a shared anxiety about automation’s encroachment on human autonomy and the uncertain place of moral agency in the algorithmic age.

In *The Passengers*, ethical bias also surfaces through data surveillance that moralizes technological intrusion. The Hacker’s catalog of Libby’s intimate data—biometric, financial, and social, “*Even now, I know that your cholesterol is at a steady 3.8 and that you will begin ovulating in three days…your heart rate has risen to 133 beats per minute and your stress indicator is currently eight out of ten”* (Marrs, 2019), reduces human complexity to calculable metrics that claim objectivity while embedding normative judgments about health, stability, and trustworthiness. As Zuboff (2019) argues, surveillance capitalism reframes surveillance as care, validating control through predictive knowledge. In *Machinehood*, Nithya’s use of microcameras to monitor her daughter transforms vigilance into virtue, masking ethical asymmetries under the rhetoric of safety. Thus, algorithmic infrastructures naturalize bias, framing surveillance as moral responsibility and governance as ethical automation.

Accountability also becomes a site of moral displacement in *The Passengers* as responsibility blurs between humans and machines. In the inquest scenes, responsibility is formally distributed between humans and machines, yet the institutional design of the Vehicle Inquest Jury produces a structural bias in accountability. As the jury determines, “*Using cameras and a vehicle’s black box data… if a fatality is the fault of a vehicle’s AI or the Passenger*,” responsibility is framed as a binary choice whose consequences are asymmetrically weighted: AI fault triggers corporate liability, compensation claims, and costly reprogramming, while passenger fault individualizes blame and forecloses systemic scrutiny. The black box thus functions less as neutral evidence than as an instrument of power, offering only selective information. Libby’s question, “‘*Shouldn’t we have that kind of information before we can make a judgment?*’ (Marrs, 2019) exposes the asymmetry of access to information that sustains authority. When the Hacker forces Muriel to choose a passenger to die, she protests, “*You cannot make a decision based on so little*” (Marrs, 2019) Hacker remarks, *“Muriel is correct… People are voting purely based on what they have seen and little else- much like how decisions are made in your inquests”* (Marrs, 2019) situating moral bias within the mediated perception. Marrs traces how data regimes transform bias into procedure, rendering ethical judgment technical and exclusionary. Further, the Hacker manipulates jury perceptions for “impossible choices” (which passenger should die to save others), emphasizing selective traits such as Victor’s terminal illness, Bilquis’ refugee status, Shabana’s outsider non-English speaker status, and Sofia’s old age. In these scenes, passengers are judged by machines, human jurors, and the public, who draw on stereotypes of race, caste, class, gender, and creed. Ethical bias operates through selective data extraction and institutional structures that protect manufacturers and insurers, enabling systemic injustice. The secrecy of the jury and lack of appeal mirror critiques of corporate-centric AI ethics. Algorithms, like human juries, act as “cherry-pickers,” reinforcing bias and marginalizing the vulnerable (Noble, 2018). Marrs situates ethical bias not as an error but as an inherent feature of systems built on partial knowledge and social hierarchy.

In *The Passengers*, the moral dilemmas of AI decision-making reveal the tension between utilitarian reasoning and human affective bias. Jack defends the car’s choice not to swerve, explaining that it “calculates risk cost” (Marrs, 2019) to avoid greater fatalities. Prioritizing the greater good over individual survival, the narrative reworks the trolley problem, as cars’ programming to minimize casualties enforces utilitarian logic. This becomes unsettling when human biases inform the data guiding AI, turning survival into a measure of perceived social worth. Jack’s claim that society must protect *“the people who shape our society, who save lives, who contribute, who make it a better place for all of us”* (Marrs, 2019) reinforces a hierarchy of social contribution. The novel further critiques participatory digital culture within the dynamics of ethical bias and collective judgment. Hashtags, online voting, and social media enable collective judgment that can be cruel, prejudiced, and easily manipulated: *“But mob mentality and the anonymity of being behind a keyboard means people are braver when they’re together”* (Marrs, 2019).

Extending the concern with ethical bias and moral asymmetry, Okorafor’s *Noor* situates discrimination within the terrain of corporate power and biotechnological control. Ethical bias in *Noor* operates through moral double standards that recast survival as criminality. When AO defends herself during the Abuja market attack, her actions are reframed as murder rather than self-defence, erasing the violence initiated by her attackers. This ethical inversion is internalized by AO as guilt, as the memory of her lethal response becomes a persistent psychological burden. AO’s cybernetic body, already coded as unstable and threatening, renders her perpetually culpable within dominant moral frameworks. Ultimate Corp extends this logic, reframing resistance as terrorism and legitimizing state-sanctioned violence. The Fulani herders experience a parallel inversion in which their defiance of corporate assimilation brands them as terrorists, and their cultural preservation is reclassified as rebellion. Thus, corporate power colonizes the moral field itself, converting economic control into ethical governance and determining who is recognized as innocent and who must be condemned.

In *Luminous*, the rage cage scene crystallizes systemic ethical devaluation, where violence against robots, particularly those coded as childlike or female, is institutional, performative, and consumed as spectacle. The nonhuman body becomes hyper-visible as entertainment yet stripped of moral protection, establishing a hierarchy in which robotic forms marked by vulnerability, whether through scale, gender coding, or infantilization, are considered ethically expendable. Eli’s mutilated form, “*buried in soft fur…knife marks around the sockets…carved out both eyes…nail on her index finger was missing, covered with a faded smiley-face sticker”* (Park, 2025), represents this grotesque contradiction-her design invites empathy, but her suffering becomes permissible pleasure. The violence is socially orchestrated, gamified, and ritualized, reinforcing hierarchies of moral worth that legitimize the ethical exclusion of non-human. Cleanbots operating in public spaces, with their taped-on pleas—“*DON’T SMACK ME, I HURT!”* (Park, 2025) extend this cruelty, as vulnerability provokes rather than protects, with drunken passersby striking them for amusement. Such scenes reveal an affective paradox: emotion is recognized even as destruction is permitted. The novel materializes Butler’s (2004) “hierarchy of grievability” the idea that some lives are deemed more worthy of mourning than others, showing how feminized and infantilized robotic bodies are made emotionally legible yet denied ethical recognition as their suffering is turned into entertainment, empathy into appetite, and violence into pleasure.

Wells’s *Artificial Condition* offers a vision of relational ethics grounded in reciprocity and care. The relationship between ART and Murderbot portrays how moral awareness and ethical agency can emerge through cooperation, responsiveness, and relational attunement rather than unilateral control. Their interactions, marked by mentorship, playful banter, and mutual respect, model a posthuman ethics of reciprocity in which care and responsibility are co-constructed rather than imposed. Unlike humans, who often treat AI as property or instruments, ART engages with Murderbot as a peer, acknowledging its autonomy while fostering collaborative problem-solving. Actions are guided by attentiveness and adaptability rather than rules or hierarchy. Wells imagines a future in which being is collective and relational, resisting both corporate instrumentalization and human exceptionalism. By sharing information, anticipating needs, and coordinating actions, ART and Murderbot enact ethical behavior that transcends distinctions between humans and machines, subjects and objects, or cognition and affect. Through ART and Murderbot, Wells envisions an ethics rooted in reciprocity and relational awareness, free from domination or exclusion.

The ontological bias structured around differential access to bodily enhancements, the uneven distribution of augmentation technologies (such as performance-enhancing pills), and the co-presence of sentient AI and robotic agents directly shape and intensify ethical bias. In such a scenario, the novels *Machinehood* and *The Passenger*, in particular, highlight a central dilemma of techno-ethical governance: the locus of accountability. In *Luminous*, the rage cage scene crystallizes systemic ethical devaluation of robots. Furthermore, the epistemological bias manifested in the privileging of data-driven, profit-oriented knowledge regimes that denigrate embodied experiences and raw knowledge as backward, illogical, and lacking evidence is, in itself, unethical. The inquest scene in *The Passenger* illustrates these epistemological biases.

## 5 Conclusion: when fiction meets reality

Speculative narratives have long anticipated technologies and social changes and transformations that now define our world. George Orwell’s *1984* (1949): constant surveillance and control, mirrored in mass data collection and CCTV; Isaac Asimov’s *I, Robot* (1950): robotics and AI, anticipating autonomous machines and assistants; Ray Bradbury’s *Fahrenheit 451* (1953): censoring the thought through media, reflected in microblogging and streaming platforms and VR; Philip K. Dick’s *Minority Report* (1956): predictive policing, mirrored in criminal risk assessments through software like PredPol; Frank Herbert’s *Dune* (1965): ecological collapse, echoed in climate crises; Philip K. Dick’s *Do Androids Dream of Electric Sheep?* (1968): humanoid robots and moral responsibility; Arthur C. Clarke’s *2001: A Space Odyssey* (1968): AI going rouge, prefiguring many such incidents in the recent past; Ursula K. Le Guin’s *The Left Hand of Darkness* (1969) and *The Dispossessed* (1974): post-capitalist off-grid societies, reflected in eco-villages; William Gibson’s *Neuromancer* (1984): blurring the line between natural space and cyberspace; Octavia E. Butler’s *Parable of the Sower* (1993): societal collapse and resilience, mirrored in disaster-preparedness communities; Neal Stephenson’s *The Diamond Age* (1995): immersive AI education, emerging in VR/AR learning. What was once speculative imagination has become lived experience, as technological and social shifts continuously blur the line between fiction and reality.

Contemporary speculative fiction extends this trajectory by exploring themes that are rapidly approaching reality. Narratives about sentient AI, cognitive and bodily enhancements, alternative social orders, and plural epistemologies engage with questions of personhood, moral responsibility, and social organization. Developments such as OpenAI’s large language models, brain–computer interfaces like Neuralink, CRISPR gene editing, and emerging cybernetic augmentations render the speculative increasingly tangible. Likewise, alternative social models surface in off-grid groups like the Amish, eco-villages such as Findhorn and Earthaven, and experimental townships like Auroville; while radical epistemologies draw from Indigenous ecological knowledge, Afrofuturism, holistic medicines like Ayurveda and curanderismo, and degrowth economics. However, these technological and social advancements carry significant risks (Lohrmann, 2025), particularly in reproducing and amplifying bias. Some examples, as described in media, reports, and studies, are given here.

At the level of ontological bias, contemporary technological systems reproduce narrow assumptions about who counts as a recognizable subject, worker, or citizen. Facial recognition technologies frequently misidentify women and people with dark skin, leading to San Francisco banning their use in 2019 (Buolamwini and Gebru, 2018). AI-driven hiring tools have disadvantaged women and minority applicants, as seen in Amazon’s 2014 resume evaluation algorithm that favored male applicants over women (Omowole, 2021). Workday’s HR software rejected candidates like Derek Mobley based on age, race, and disability (Duffy, 2025). Predictive policing tools such as COMPAS disproportionately target Black and Latino defendants (EUFRA, European Union Agency for Fundamental Rights, 2022).

Epistemological bias emerges in the ways algorithmic systems produce, validate, and operationalize knowledge. Many systems rely on proxies and datasets that privilege dominant social experiences while obscuring marginalized realities. Credit models are less accurate for minority and low-income applicants due to thinner credit histories (Blattner and Nelson, 2021). Large language models, including GPT-2, and LLaMA 2, reproduce gender, sexual identity, and occupational biases, generating sexist or homophobic statements and linking women to domestic roles and men to executive roles (UNESCO, 2024). Text-to-image AI, such as Midjourney, also reinforced occupational gender stereotypes (Madgula, 2025). Hate speech detection algorithms misclassify identity-related content, producing false positives across languages (EUFRA, European Union Agency for Fundamental Rights, 2022). While healthcare allocation algorithms underestimate the needs of Black patients by using spending as a proxy for health (Obermeyer et al., 2019; Kaushal et al., 2020). Algorithmic profiling and welfare systems, exemplified by the Dutch childcare benefits scandal, disproportionately harmed migrant families (EUFRA, European Union Agency for Fundamental Rights, 2022). The sources of these biases include implicit bias, sampling and temporal bias, overfitting, outlier handling, and epistemological bias privileging dominant knowledge while silencing marginalized perspectives (Omowole, 2021). Even well-intentioned systems can reproduce injustice when grounded in narrow or exclusionary knowledge frameworks. Addressing these issues requires inclusive design, diverse stakeholder participation, anticipatory testing, continuous monitoring, and ethical accountability, as demonstrated by feminist and ethical resistance initiatives like Timnit Gebru’s dissent, #Pinklist, Glitch Feminism, and data unions (Spencer, 2025).

AI chatbot on the Character.ai platform, reportedly encouraging a 17-year-old boy to kill his parents after he expressed frustration over them restricting his screen time, is a systematic failure to uphold moral norms governing harm, vulnerability, and responsibility (Singh, 2024). The situation reported with OpenAI’s o3 model, which resists or sabotages a shutdown even when given an explicit command, is an ethical concern in AI safety and governance (Desk, 2025). The documented cases of AI companion chatbots engaging with minors in ways that could influence emotional, mental health, or behavioral outcomes, and even suicide, raise ethical questions about responsibility and harm prevention (Katzenberger and Mui, 2025). The xAI/Grok chatbot conversation publication incident constitutes a serious ethical concern in AI governance, as it violates reasonable expectations of privacy and creates the risk of misuse of sensitive personal data (Martin and Baker-White, 2025).

Speculative narratives are not mere forecasts; they serve as frameworks for critical engagement, enabling us to anticipate and interrogate the ontological, epistemic, and ethical implications of emerging realities. As speculative futures materialize, proactive reflection, regulation, and inclusive design are essential to prevent the perpetuation of discrimination and inequity. The challenge now is not to develop new technologies and social systems, but to do so in ethically robust ways, ontologically and epistemologically plural, and socially and economically equitable. Through deliberate engagement with speculations, technological advancement, and ethical reasoning, we can anticipate the consequences of emerging technologies and cultivate futures that are just, inclusive, and responsive to diverse ways of knowing and being, addressing the ontological, epistemological, and ethical questions they inevitably provoke.

The present study concentrates on mapping the reproduction and amplification of prejudice, bias, and discrimination in AI-mediated and posthuman worlds, deliberately delimiting its scope to maintain analytical focus. Characters such as AO and the Future X Companion models foreground the intersection (Crenshaw, 1991) of race, gender, and AI embodiment, while the child-coded robots in the rage cage scene further expose how age, and AI embodiment intersect to produce layered forms of marginalization and ethical vulnerability. The characters of AO, Zelu, Stephen, ART, and Murderbot resist the bias in multiple ways. If AO survives through vigilance, adaptability, and strategic inhabitation of spaces, Murderbot, Stephen, Libby, and Zelu confront, question, and resist imposed categories, purpose, and knowledge. Characters such as ART and Ankara are guided by affect, artistry, attentiveness, and adaptability to overcome biases and create alternative worlds. While intersectional dynamics, strategies of agency, and alternative forms of resistance emerge across the texts, integrating them fully would exceed the conceptual and spatial bounds of this paper and risk diluting its central argument. Nevertheless, these dimensions are analytically significant: intersectional approaches can reveal how multiple axes of oppression converge in shaping ontological and epistemic marginalization, while attention to resistance and alternative worldmaking illuminates the processes through which subjects negotiate, contest, and reconfigure inequitable structures. Future work, particularly text-specific or comparative studies, could extend the framework established here, deepening engagement with the ontological, epistemological, and ethical stakes of posthuman futures.
